# Correction: Healthcare professionals’ perceptions about implementing accreditation as a strategy to improve healthcare quality and organisational performance: a cross-sectional survey study

**DOI:** 10.1371/journal.pone.0331388

**Published:** 2025-09-02

**Authors:** Mohammad J. Alhawajreh, William J. Jackson, Audrey S. Paterson

There is an error in affiliation 1 for author Mohammad J. Alhawajreh. The correct affiliation 1 is: Health Services Management, Middle East University, Amman, Jordan,
