# Supplementary material for: Healthcare professionals’ perceptions about implementing accreditation as a strategy to improve healthcare quality and organisational performance: a cross-sectional survey study
Source: PLoS One. 2025 Mar 25;20(3):e0320664. doi: 10.1371/journal.pone.0320664 (PMC11936192; doi:10.1371/journal.pone.0320664)
Supplement: S1 Appendix — (DOCX) [file pone.0320664.s001.docx]

**Supporting information appendices**

**S1 Appendix**

**The main characteristics of the case study sites.**

| **Case / Characteristic** | **Early Accredited Sites** | | **Late Accredited Sites** | |
| --- | --- | --- | --- | --- |
|  | **Case 1** | **Case 2** | **Case 3** | **Case 4** |
| **Region** | Central | North | South | Central |
| **Governorate** | A | C | D | E |
| **Type of hospital** | Academic public hospital | Academic Public hospital | Academic public hospital | Academic public hospital |
| **Hospital size** | A large-sized | A medium -sized | A medium-sized | A large-sized |
| **Number of beds** | Approximately 443 beds | Over 130 beds | Around 172 beds | Approximately 494 |
| **Number of employees** | 609 staff | 395 staff | 800 staff | 1,136 staff |
| **Accreditation Status** | Accredited for many years | Accredited for many years | Newly accredited | Accredited for the first time |
| **Length of participation in**  **accreditation** | Since 2011 | Since 2011 | Since late 2019 | Since 2020 |
| **Availability of quality/Accreditation unit** | Available | Available | Available | Available |
| **Type of accreditation** | Voluntary, HCAC hospital accreditation program | Voluntary, HCAC hospital accreditation program | Voluntary, HCAC hospital accreditation program | Voluntary, HCAC hospital accreditation program |

Source: HCAC (2021); MOH (2020, 2021)

**S2 Appendix**

SQUIRE guidelines for reporting system-level work (accreditation) to improve the quality, safety, and value of healthcare.

| **Title and Abstract** | **Page Number** |
| --- | --- |
| **Title**: Indicate that the manuscript concerns an initiative to improve healthcare (broadly defined to include the quality, safety, effectiveness, patient-centeredness, timeliness, cost, efficiency, and equity of healthcare) | 1 |
| **Abstract**:   1. Provide adequate information to aid in searching and indexing 2. Summarize all key information from various sections of the text using the abstract format of the intended publication or a structured summary such as: background, local [problem](http://squire-statement.org/index.cfm?fuseaction=page.viewpage&pageid=485#Problem), methods, interventions, results, conclusions | 2 |

| **Introduction** | **Page Number** |
| --- | --- |
| **Problem**: Nature and significance of the local [problem](http://squire-statement.org/index.cfm?fuseaction=page.viewpage&pageid=485#Problem) | **4 - 5** |
| **Available Knowledge**: Summary of what is currently known about the [problem](http://squire-statement.org/index.cfm?fuseaction=page.viewpage&pageid=485#Problem), including relevant previous studies | **4 - 5** |
| **Rationale**: Informal or formal frameworks, models, concepts, and/or [theories](http://squire-statement.org/index.cfm?fuseaction=page.viewpage&pageid=485#Theory) used to explain the [problem](http://squire-statement.org/index.cfm?fuseaction=page.viewpage&pageid=485#Problem), any reasons or [assumptions](http://squire.citysoft.org/index.cfm?fuseaction=page.viewPage&pageID=485&nodeID=1#assumptions) that were used to develop the [intervention(s),](http://squire-statement.org/index.cfm?fuseaction=page.viewpage&pageid=485#Interventions) and reasons why the [intervention(s)](http://squire-statement.org/index.cfm?fuseaction=page.viewpage&pageid=485#Interventions) was expected to work | **-** |
| **Specific Aims**: Purpose of the project and this report | **5** |

| **Methods** | **Page Number** |
| --- | --- |
| **Context**: Contextual elements considered important at the outset of introducing the intervention(s) | **6 - 7** |
| **Intervention(s):**   1. Description of the intervention(s) in sufficient detail that others could reproduce it 2. Specifics of the team involved in the work | **6 - 7** |
| **Study of the Intervention(s):**   1. Approach chosen for assessing the impact of the intervention(s) 2. Approach used to establish whether the observed outcomes were due to the intervention(s) | **8** |
| **Measures**:   1. Measures chosen for studying processes and outcomes of the intervention(s), including rationale for choosing them, their operational definitions, and their validity and reliability 2. Description of the approach to the ongoing assessment of contextual elements that contributed to the success, failure, efficiency, and cost 3. Methods employed for assessing completeness and accuracy of data | **8 - 9** |
| **Analysis**:   1. Qualitative and quantitative methods used to draw inferences from the data 2. Methods for understanding variation within the data, including the effects of time as a variable | **9** |
| **Ethical Considerations**: [Ethical aspects](http://squire-statement.org/index.cfm?fuseaction=page.viewpage&pageid=485#Ethical_aspects)of implementing and studying the [intervention(s)](http://squire-statement.org/index.cfm?fuseaction=page.viewpage&pageid=485#Interventions) and how they were addressed, including, but not limited to, formal ethics review and potential conflict(s) of interest | **7** |

| **Results** | **Page Number** |
| --- | --- |
| **Result**:   1. Initial steps of the intervention(s) and their evolution over time (e.g., time-line diagram, flow chart, or table), including modifications made to the intervention during the project 2. Details of the process measures and outcome 3. Contextual elements that interacted with the intervention(s) 4. Observed associations between outcomes, interventions, and relevant contextual elements 5. Unintended consequences such as unexpected benefits, problems, failures, or costs associated with the intervention(s). 6. Details about missing data | **11 - 17** |

| **Discussion** | **Page Number** |
| --- | --- |
| **Summary**:   1. Key findings, including relevance to the rationale and specific aims 2. Particular strengths of the project | **18** |
| **Interpretation**:   1. Nature of the association between the intervention(s) and the outcomes 2. Comparison of results with findings from other publications 3. Impact of the project on people and systems 4. Reasons for any differences between observed and anticipated outcomes, including the influence of context 5. Costs and strategic trade-offs, including opportunity costs | **18 - 22** |
| **Limitations**:   1. Limits to the generalizability of the work 2. Factors that might have limited internal validity such as confounding, bias, or imprecision in the design, methods, measurement, or analysis 3. Efforts made to minimize and adjust for limitations | **22** |
| **Conclusions**:   1. Usefulness of the work 2. Sustainability 3. Potential for spread to other contexts 4. Implications for practice and for further study in the field 5. Suggested next steps | **23** |

| **Other** | **Page Number** |
| --- | --- |
| **Funding**: Sources of funding that supported this work. Role, if any, of the funding organization in the design, implementation, interpretation, and reporting | **32** |

**S3 Appendix**

**Minimum sample size and number of questionnaires that needed for each case in the study.**

| **Characteristics / Case** | **Actual number of employees** | **Eligible employees to participate in the survey (Sample frame)** | **Percentage (%) of Sample frame** | **Minimum sample size needed*** | **Number of distributed questionnaires (Minimum sample size + 50%)** |
| --- | --- | --- | --- | --- | --- |
| **Case 1** | 609 | 410 | 20.47 | 68 | 102 |
| **Case 2** | 395 | 224 | 11.18 | 37 | 56 |
| **Case 3** | 800 | 572 | 28.57 | 95 | 142 |
| **Case 4** | 1,136 | 796 | 39.78 | 133 | 200 |
| **Total** | 2940 staff | 2002 staff | 100 % | 333 staff | 500 questionnaires |
| *This sample is based on a margin of error of (+/–5%), a confidence level of 95%, and a population percentage or variability of 50% (Fowler, 2009). | | | | | |

**S4 Appendix:**

**A copy of the questionnaire.**


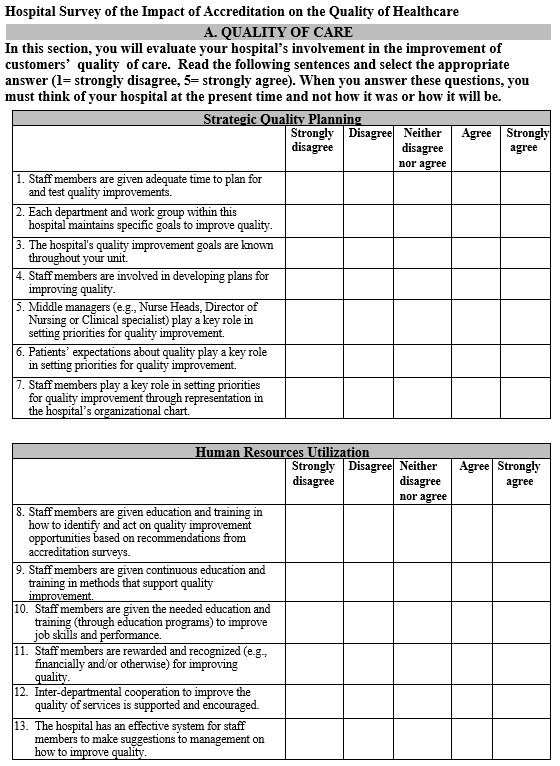


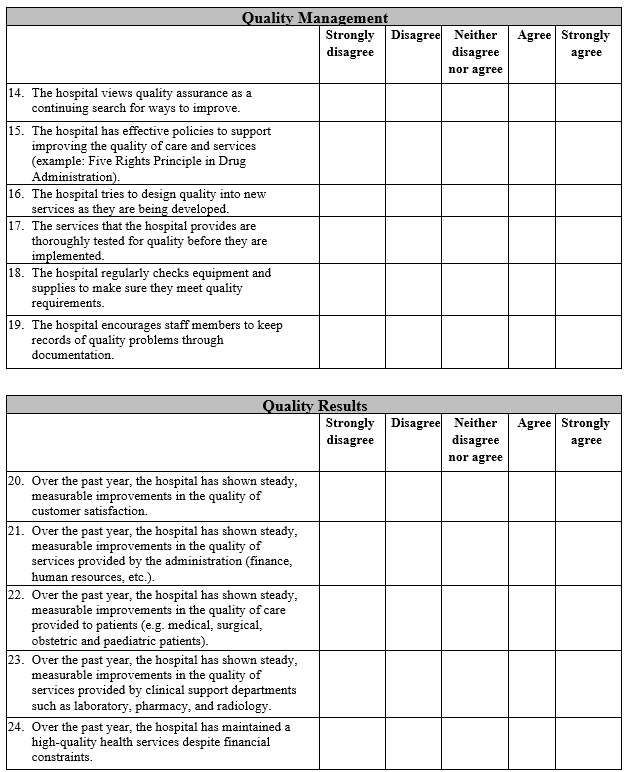


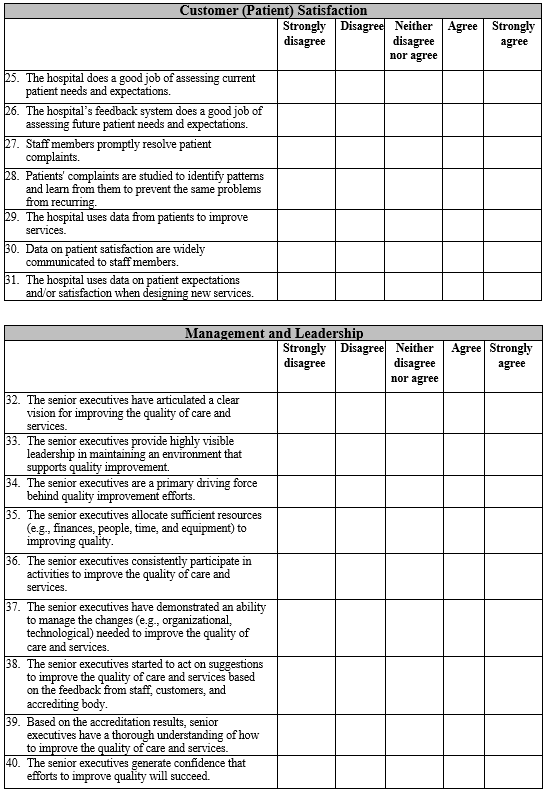


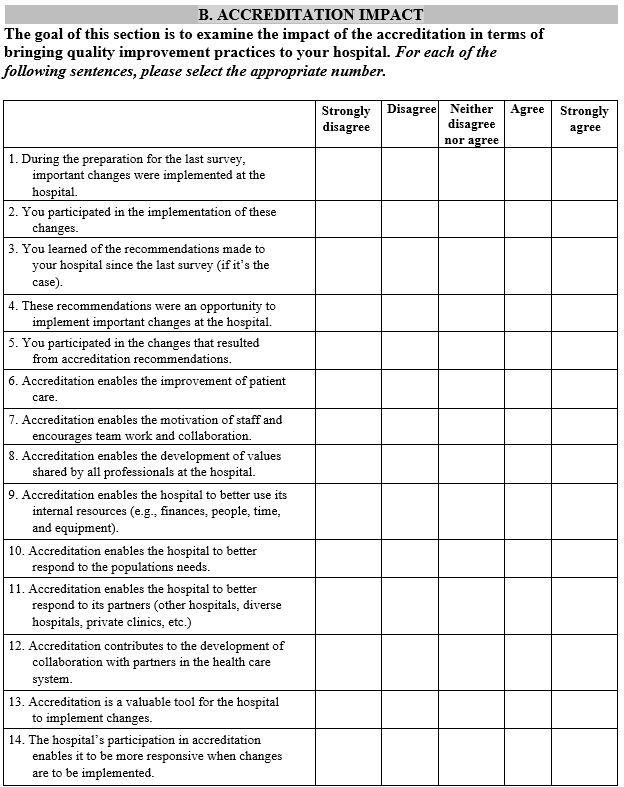


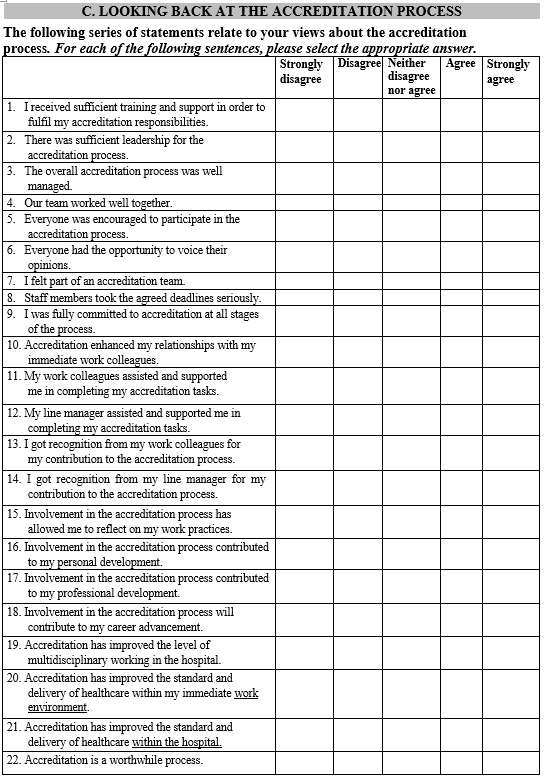


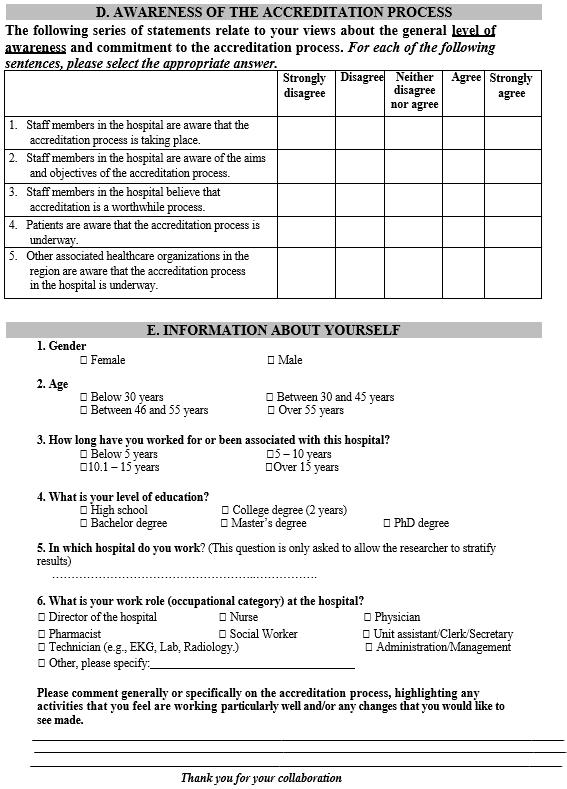


**S5 Appendix**

**Participant’s responses to the accreditation survey’s various scales and subscales.**


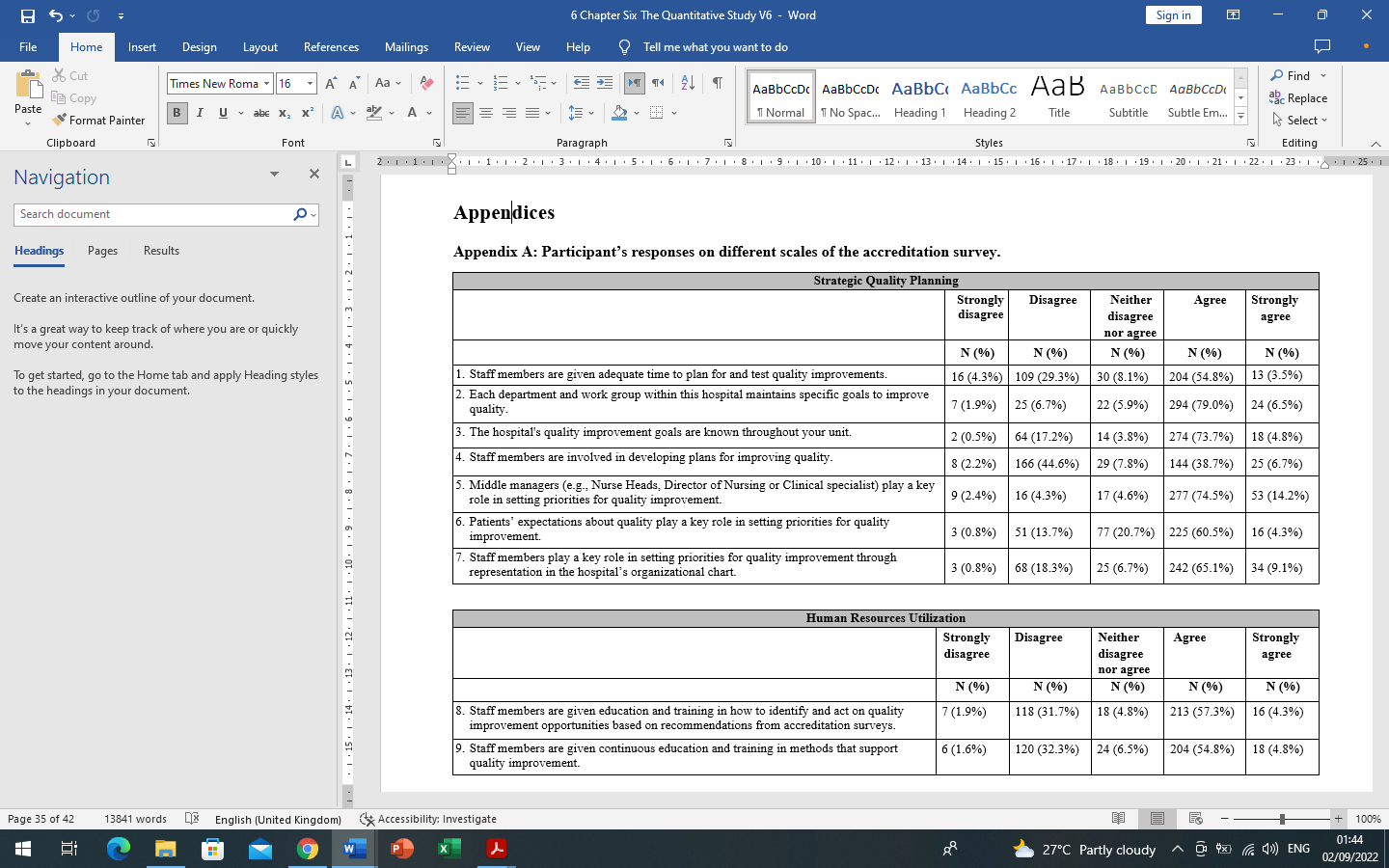


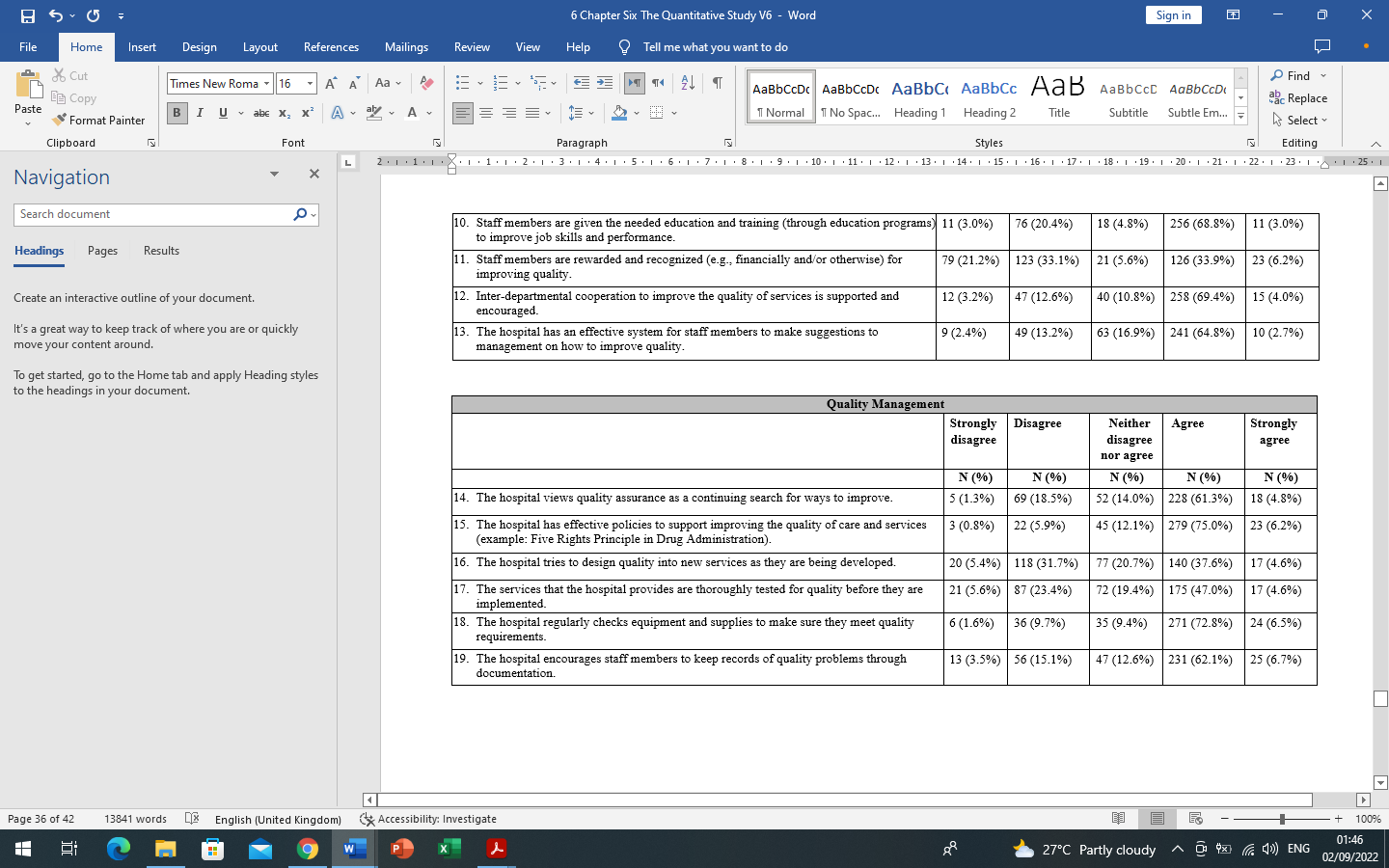


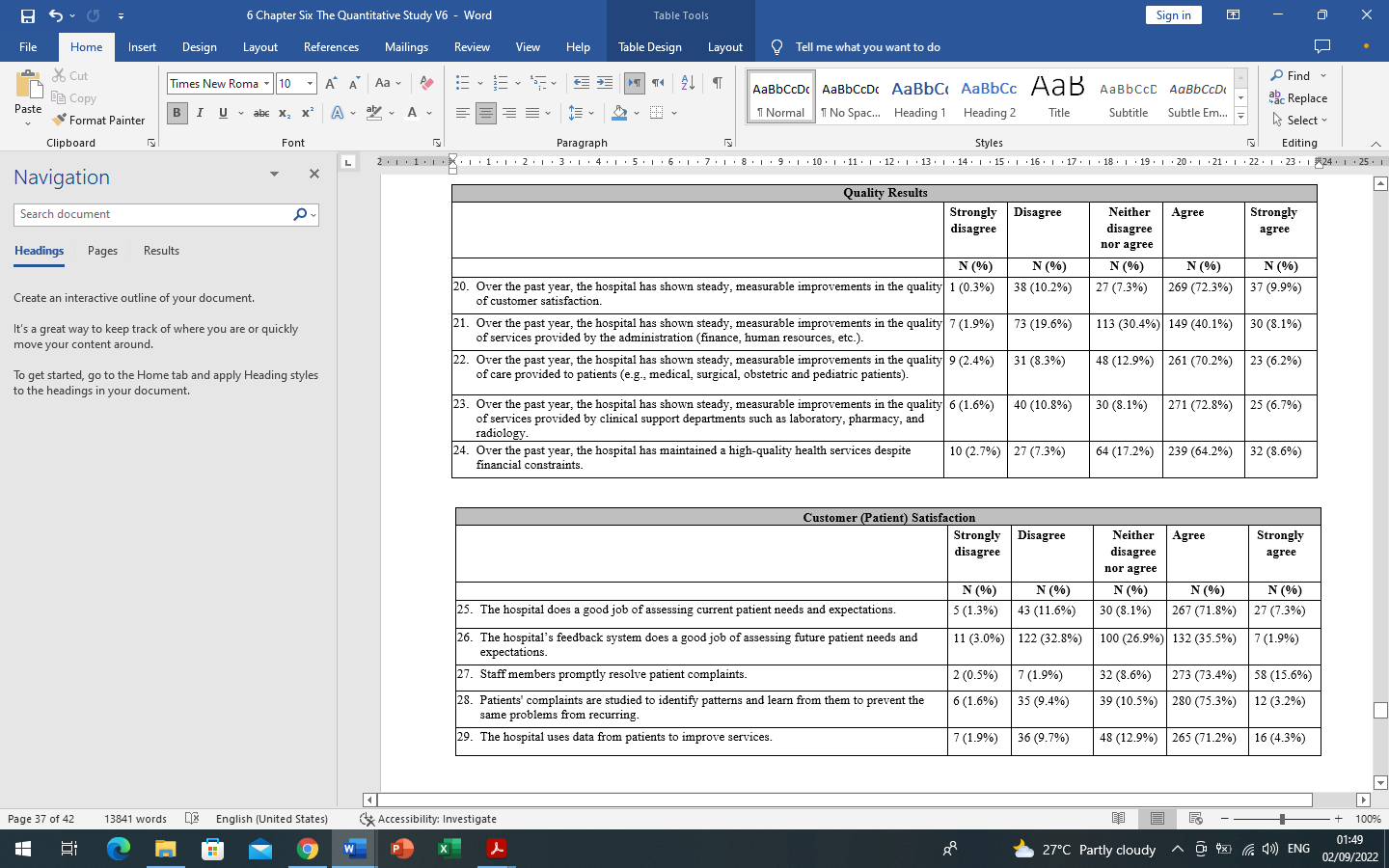


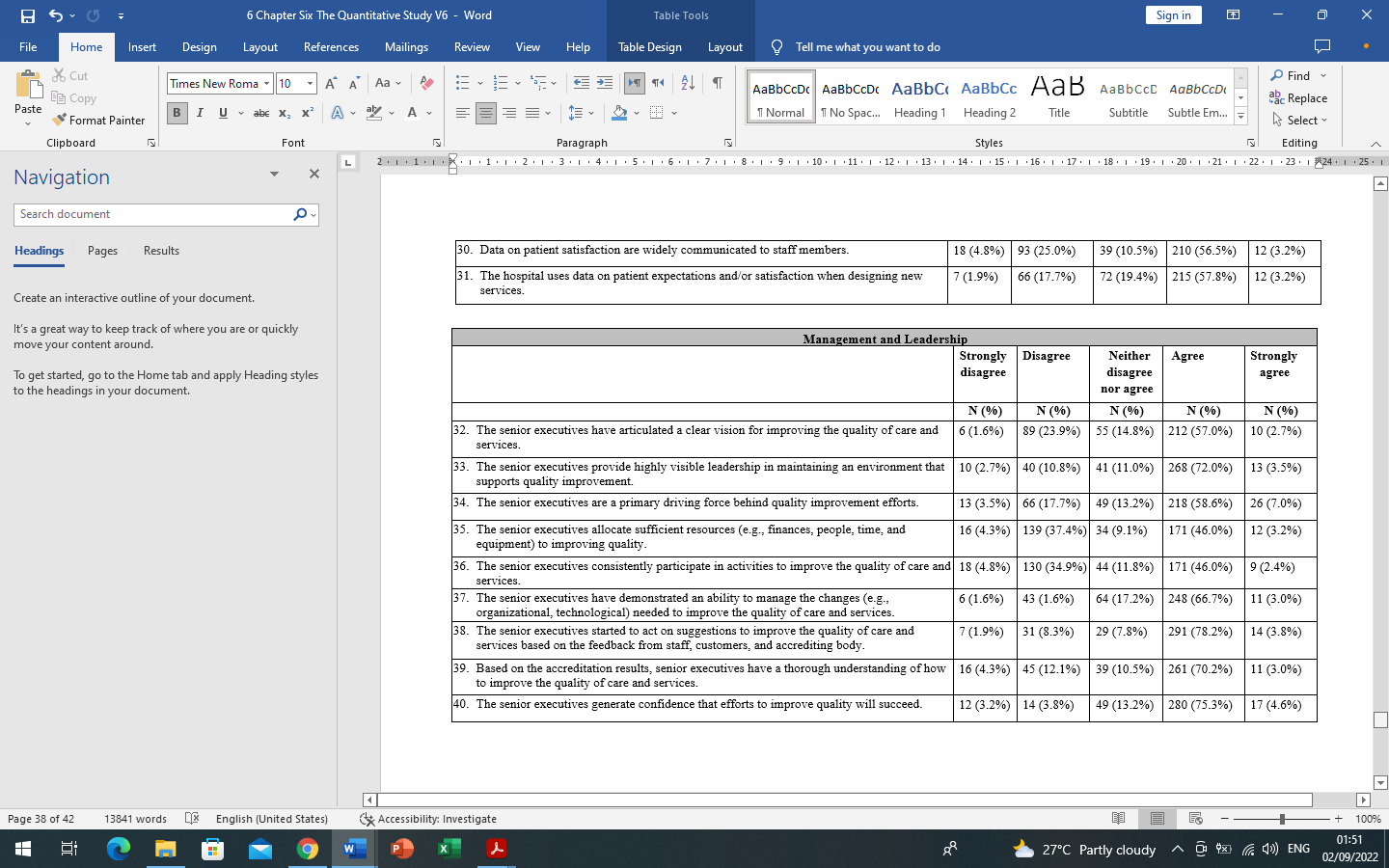


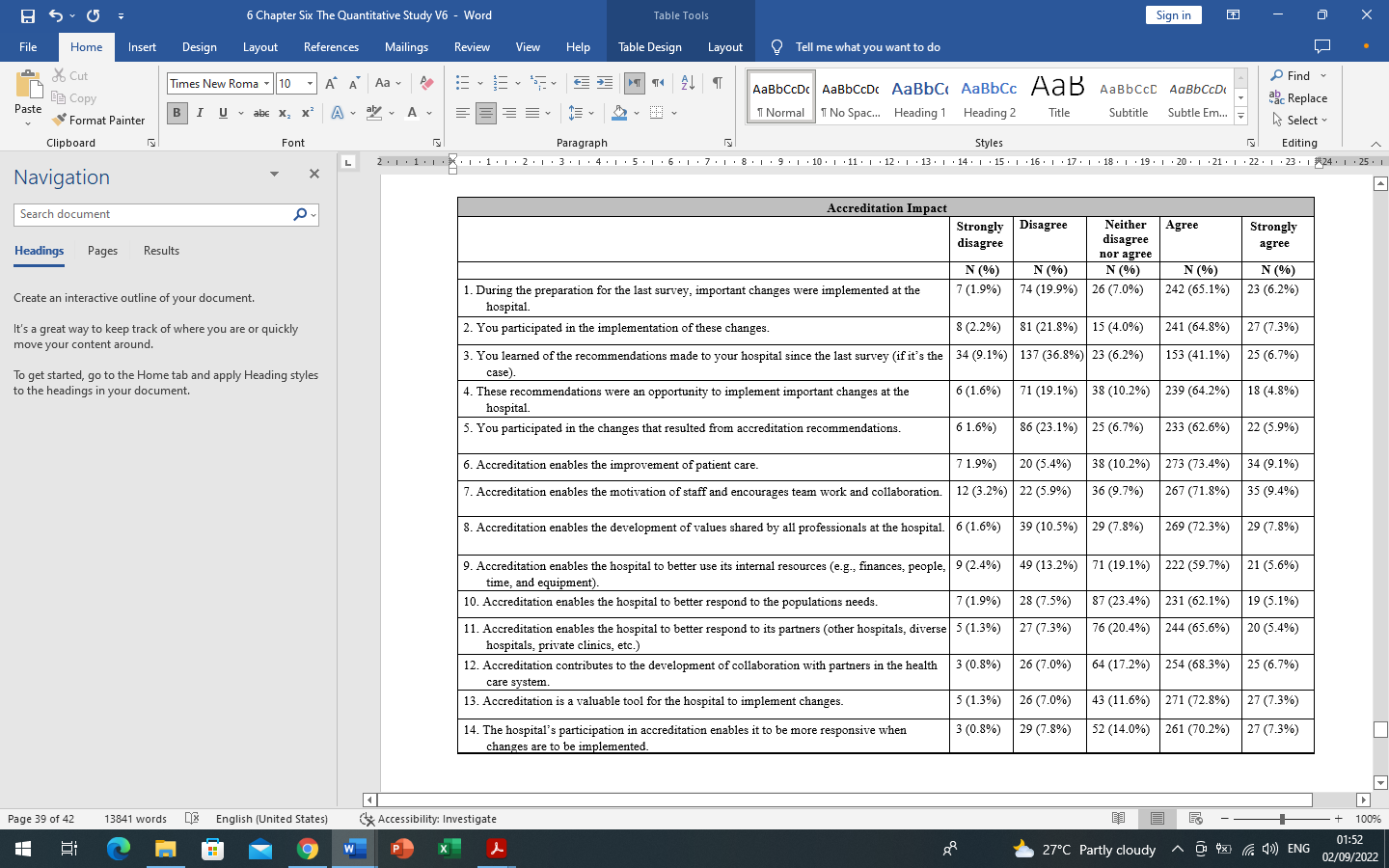


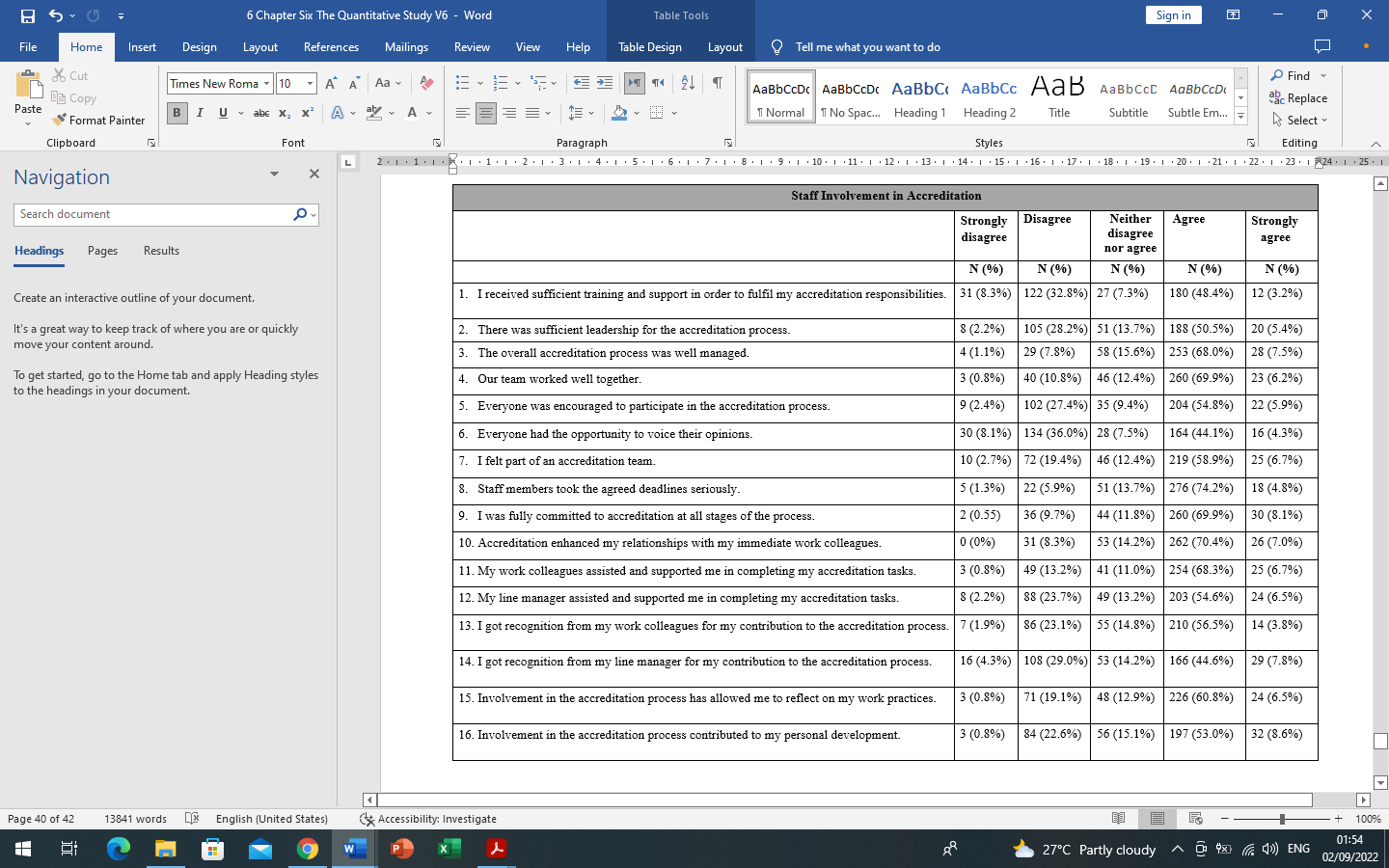


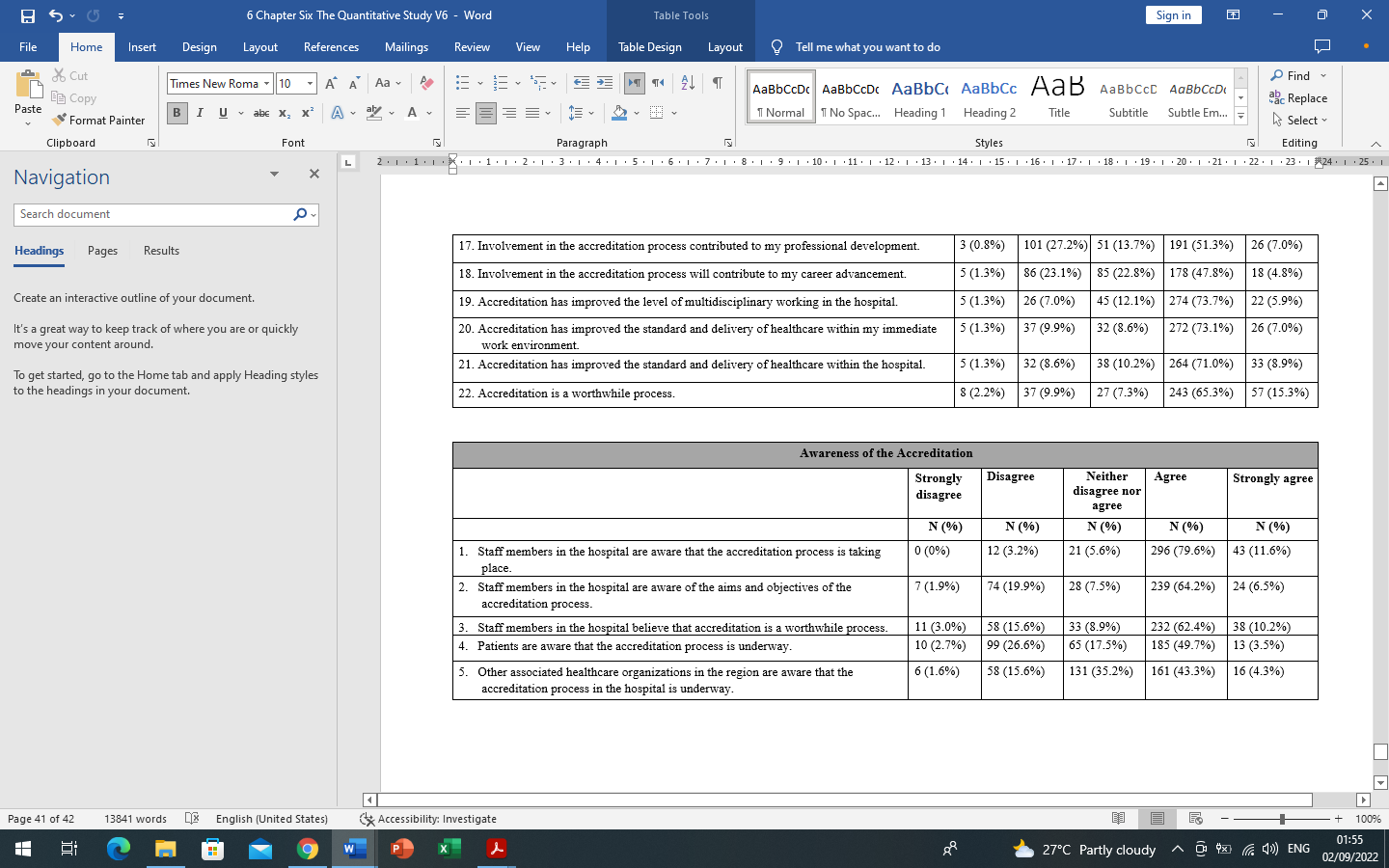


**S6 Appendix**

**Result of significant and non-significant associations of each survey scale with demographic variables.**

| **Scale** | **Gender** | **Age** | **Working experience** | **Level of education** | **Occupational categories** | **Hospital size** |
| --- | --- | --- | --- | --- | --- | --- |
| Strategic Quality Planning | NS | **S** | **S** | **S** | NS | NS |
| Human Resource Utilization | NS | **S** | **S** | **S** | **S** | NS |
| Quality Management | **S** | **S** | **S** | S | **S** | NS |
| Quality Results | NS | **S** | **S** | **S** | NS | NS |
| Customer or Patient Satisfaction | **S** | **S** | **S** | **S** | **S** | NS |
| Management and Leadership | NS | **S** | **S** | **S** | S | NS |
| Accreditation |  | | | | | |
| Accreditation Impact | **NS** | **S** | **S** | **S** | NS | NS |
| Staff Involvement in Accreditation | NS | **S** | **S** | **S** | **S** | NS |
| Awareness of the Accreditation | NS | **S** | **S** | **S** | **S** | NS |
| **S**: Significant, **NS**: Non-significant | | | | | | |

**S7 Appendix** **(Table 1- Table 9)**

**Analysis of mean ranks for each scale of the survey with the demographic variables.**

**Table 1 Strategic Quality Planning Scores in Relation to Demographics**

|  | **N** | **Mean Rank** | **Kruskal Wallis (df)** | **P-Value** |
| --- | --- | --- | --- | --- |
| **Gender**  Male  Female | 152  220 | 184.78  187.69 | 0.067(1) | 0.796 |
| **Age of respondents**  < 30 years  30 – 45 years  46 – 55 years  > 55 years | 118  187  41  26 | 153.86  186.21  256.51 *  226.33 | 32.281(3) | **< 0.001** |
| **Working experience in years**  3 – <5  5 – 10  >10 – <15  > 15 | 95  116  100  61 | 147.54  174.80  204.27  240.30 * | 32.310(3) | **< 0.001** |
| **Level of education**  Diploma (2 years)  Bachelor’s degree  Master’s and PhD degree | 66  252  54 | 220.85 *  173.48  205.28 | 12.251(2) | **0.002** |
| **Occupational categories**  Director of the hospital  Nurse  Physician  Pharmacist  Social Worker  Allied health technician  Administration/Management | 12  144  86  19  7  43  61 | 233.92  197.14  167.90  173.74  154.36  169.55  197.90 | 9.095(6) | 0.168 |
| **Hospital size**  Medium  Large | 149  223 | 193.91  181.55 | 1.197(1) | 0.274 |
| *. Indicating to statistically significant difference based on Mann-Whitney test. | | | | |

**Table 2 Human Resource Utilization Scores in Relation to Demographics**

|  | **N** | **Mean Rank** | **Kruskal Wallis (df)** | **P-Value** |
| --- | --- | --- | --- | --- |
| **Gender**  Male  Female | 152  220 | 190.25  183.91 | 0.319(1) | 0.572 |
| **Age of respondents**  < 30 years  30 – 45 years  46 – 55 years  > 55 years | 118  187  41  26 | 148.54  191.67  245.96 *  227.83 | 32.193(3) | **< 0.001** |
| **Working experience in years**  3 – <5  5 – 10  >10 – <15  > 15 | 95  116  100  61 | 152.08  175.70  201.03  236.81 * | 26.640(3) | **< 0.001** |
| **Level of education**  Diploma (2 years)  Bachelor’s degree  Master’s and PhD degree | 66  252  54 | 220.80 *  176.01  193.54 | 9.547(2) | **0.008** |
| **Occupational categories**  Director of the hospital  Nurse  Physician  Pharmacist  Social Worker  Allied health technician  Administration/Management | 12  144  86  19  7  43  61 | 256.96 *  184.17  181.88  170.13  152.50  151.31  218.44 | 16.859(6) | **0.010** |
| **Hospital size**  Medium  Large | 149  223 | 191.69  183.03 | 0.593(1) | 0.441 |
| *. Indicating to statistically significant difference based on Mann-Whitney test. | | | | |

**Table 3 Quality Management Scores in Relation to Demographics**

|  | **N** | **Mean Rank** | **Kruskal Wallis (df)** | **P-Value** |
| --- | --- | --- | --- | --- |
| **Gender**  Male  Female | 152  220 | 213.21 *  168.05 | 16.266(1) | **< 0.001** |
| **Age of respondents**  < 30 years  30 – 45 years  46 – 55 years  > 55 years | 118  187  41  26 | 149.02  197.08  223.60 *  222.04 | 24.486(3) | **< 0.001** |
| **Working experience in years**  3 – <5  5 – 10  >10 – <15  > 15 | 95  116  100  61 | 144.86  175.94  213.28  227.55 * | 31.246(3) | **< 0.001** |
| **Level of education**  Diploma (2 years)  Bachelor’s degree  Master’s and PhD degree | 66  252  54 | 203.97  177.00  209.46 * | 6.330(2) | **0.042** |
| **Occupational categories**  Director of the hospital  Nurse  Physician  Pharmacist  Social Worker  Allied health technician  Administration/Management | 12  144  86  19  7  43  61 | 268.17 *  193.93  170.23  157.95  194.93  142.98  214.45 | 22.700(6) | **< 0.001** |
| **Hospital size**  Medium  Large | 149  223 | 191.04  183.47 | 0.454(1) | 0.500 |
| *. Indicating to statistically significant difference based on Mann-Whitney test. | | | | |

**Table 4 Quality Results Scores in Relation to Demographics**

|  | **N** | **Mean Rank** | **Kruskal Wallis (df)** | **P-Value** |
| --- | --- | --- | --- | --- |
| **Gender**  Male  Female | 152  220 | 186.04  186.82 | 0.005(1) | 0.944 |
| **Age of respondents**  < 30 years  30 – 45 years  46 – 55 years  > 55 years | 118  187  41  26 | 154.11  186.53  241.35  246.77 * | 30.994(3) | **< 0.001** |
| **Working experience in years**  3 – <5  5 – 10  >10 – <15  > 15 | 95  116  100  61 | 140.98  184.67  194.48  247.78 * | 39.250(3) | **< 0.001** |
| **Level of education**  Diploma (2 years)  Bachelor’s degree  Master’s and PhD degree | 66  252  54 | 219.64  170.24  221.88 * | 18.756(2) | **< 0.001** |
| **Occupational categories**  Director of the hospital  Nurse  Physician  Pharmacist  Social Worker  Allied health technician  Administration/Management | 12  144  86  19  7  43  61 | 212.25  188.70  176.62  140.24  191.93  189.64  201.73 | 6.577(6) | 0.362 |
| **Hospital size**  Medium  Large | 149  223 | 188.32  185.28 | 0.075(1) | 0.784 |
| *. Indicating to statistically significant difference based on Mann-Whitney test. | | | | |

**Table 5 Customer or Patient Satisfaction Scores in Relation to Demographics**

|  | **N** | **Mean Rank** | **Kruskal Wallis (df)** | **P-Value** |
| --- | --- | --- | --- | --- |
| **Gender**  Male  Female | 152  220 | 171.13  197.12 * | 5.336(1) | **0.021** |
| **Age of respondents**  < 30 years  30 – 45 years  46 – 55 years  > 55 years | 118  187  41  26 | 159.68  185.21  242.93 *  228.54 | 23.01(3) | **< 0.001** |
| **Working experience in years**  3 – <5  5 – 10  >10 – <15  > 15 | 95  116  100  61 | 133.92  191.09  208.95  222.86 * | 34.835(3) | **< 0.001** |
| **Level of education**  Diploma (2 years)  Bachelor’s degree  Master’s and PhD degree | 66  252  54 | 210.46  170.19  233.31 * | 19.634(2) | **< 0.001** |
| **Occupational categories**  Director of the hospital  Nurse  Physician  Pharmacist  Social Worker  Allied health technician  Administration/Management | 12  144  86  19  7  43  61 | 237.00  193.38  152.66  180.53  281.21 *  171.01  209.95 | 21.392(6) | **0.002** |
| **Hospital size**  Medium  Large | 149  223 | 189.25  184.66 | 0.166(1) | 0.684 |
| *. Indicating to statistically significant difference based on Mann-Whitney test. | | | | |

**Table 6 Management and Leadership Scores in Relation to Demographics**

|  | **N** | **Mean Rank** | **Kruskal Wallis (df)** | **P-Value** |
| --- | --- | --- | --- | --- |
| **Gender**  Male  Female | 152  220 | 180.09  190.93 | 0.931(1) | 0.335 |
| **Age of respondents**  < 30 years  30 – 45 years  46 – 55 years  > 55 years | 118  187  41  26 | 167.72  183.56  234.21 *  217.63 | 14.239(3) | **< 0.001** |
| **Working experience in years**  3 – <5  5 – 10  >10 – <15  > 15 | 95  116  100  61 | 148.83  173.50  209.16  232.74 * | 31.246(3) | **< 0.001** |
| **Level of education**  Diploma (2 years)  Bachelor’s degree  Master’s and PhD degree | 66  252  54 | 207.83  173.87  219.37 * | 11.318(2) | **0.003** |
| **Occupational categories**  Director of the hospital  Nurse  Physician  Pharmacist  Social Worker  Allied health technician  Administration/Management | 12  144  86  19  7  43  61 | 221.33  189.07  165.38  191.95  288.21 *  173.86  198.89 | 12.593(6) | **0.050** |
| **Hospital size**  Medium  Large | 149  223 | 190.14  184.07 | 0.291(1) | 0.590 |
| *. Indicating to statistically significant difference based on Mann-Whitney test. | | | | |

**Table 7 Accreditation Impact Scores in Relation to Demographics**

|  | **N** | **Mean Rank** | **Kruskal Wallis (df)** | **P-Value** |
| --- | --- | --- | --- | --- |
| **Gender**  Male  Female | 152  220 | 175.13  194.36 | 2.933(1) | 0.087 |
| **Age of respondents**  < 30 years  30 – 45 years  46 – 55 years  > 55 years | 118  187  41  26 | 140.10  198.16  253.57 *  207.46 | 41.954(3) | **< 0.001** |
| **Working experience in years**  3 – <5  5 – 10  >10 – <15  > 15 | 95  116  100  61 | 126.86  180.45  215.57  243.25 * | 54.990(3) | **< 0.001** |
| **Level of education**  Diploma (2 years)  Bachelor’s degree  Master’s and PhD degree | 66  252  54 | 208.20  170.03  236.84 * | 20.853(2) | **< 0.001** |
| **Occupational categories**  Director of the hospital  Nurse  Physician  Pharmacist  Social Worker  Allied health technician  Administration/Management | 12  144  86  19  7  43  61 | 210.17  195.81  167.28  192.61  221.86  161.01  198.98 | 8.637(6) | 0.195 |
| **Hospital size**  Medium  Large | 149  223 | 190.86  183.59 | 0.417(1) | 0.518 |
| *. Indicating to statistically significant difference based on Mann-Whitney test. | | | | |

**Table 8 Staff Involvement in Accreditation Scores in Relation to Demographics**

|  | **N** | **Mean Rank** | **Kruskal Wallis (df)** | **P-Value** |
| --- | --- | --- | --- | --- |
| **Gender**  Male  Female | 152  220 | 175.64  194.00 | 2.627(1) | 0.105 |
| **Age of respondents**  < 30 years  30 – 45 years  46 – 55 years  > 55 years | 118  187  41  26 | 126.46  202.36  266.61 *  218.63 | 66.081(3) | **< 0.001** |
| **Working experience in years**  3 – <5  5 – 10  >10 – <15  > 15 | 95  116  100  61 | 127.36  165.50  230.17  246.93 * | 69.070(3) | **< 0.001** |
| **Level of education**  Diploma (2 years)  Bachelor’s degree  Master’s and PhD degree | 66  252  54 | 223.20  167.89  228.49 * | 23.526(2) | **< 0.001** |
| **Occupational categories**  Director of the hospital  Nurse  Physician  Pharmacist  Social Worker  Allied health technician  Administration/Management | 12  144  86  19  7  43  61 | 211.38  204.61  157.07  224.63  232.29 *  149.12  189.57 | 20.119(6) | **0.003** |
| **Hospital size**  Medium  Large | 149  223 | 186.89  186.24 | 0.033(1) | 0.955 |
| *. Indicating to statistically significant difference based on Mann-Whitney test. | | | | |

**Table 9 Awareness of the Accreditation Scores in Relation to Demographics**

|  | **N** | **Mean Rank** | **Kruskal Wallis (df)** | **P-Value** |
| --- | --- | --- | --- | --- |
| **Gender**  Male  Female | 152  220 | 195.62  180.20 | 1.892(1) | 0.169 |
| **Age of respondents**  < 30 years  30 – 45 years  46 – 55 years  > 55 years | 118  187  41  26 | 128.60  203.25  264.57 *  205.69 | 62.596(3) | **< 0.001** |
| **Working experience in years**  3 – <5  5 – 10  >10 – <15  > 15 | 95  116  100  61 | 124.42  171.02  222.78  253.16 * | 70.481(3) | **< 0.001** |
| **Level of education**  Diploma (2 years)  Bachelor’s degree  Master’s and PhD degree | 66  252  54 | 218.48  166.01  243.03 * | 30.601(2) | **< 0.001** |
| **Occupational categories**  Director of the hospital  Nurse  Physician  Pharmacist  Social Worker  Allied health technician  Administration/Management | 12  144  86  19  7  43  61 | 230.92  206.85  167.52  179.76  271.93 *  135.05  185.05 | 24.792(6) | **< 0.001** |
| **Hospital size**  Medium  Large | 149  223 | 184.63  187.63 | 0.077(1) | 0.782 |
| *. Indicating to statistically significant difference based on Mann-Whitney test. | | | | |
